# DNA barcoding of fish fauna from low order streams of Tapajós River basin

**DOI:** 10.1371/journal.pone.0209430

**Published:** 2018-12-21

**Authors:** Karen Larissa Auzier Guimarães, Marcos Paulo Alho de Sousa, Frank Raynner Vasconcelos Ribeiro, Jorge Ivan Rebelo Porto, Luís Reginaldo Ribeiro Rodrigues

**Affiliations:** 1 Laboratório de Genética & Biodiversidade, Instituto de Ciências da Educação, Universidade Federal do Oeste do Pará, Santarém, Pará, Brazil; 2 Programa de Pós Graduação em Recursos Naturais da Amazônia, Universidade Federal do Oeste do Pará, Santarém, Pará, Brazil; 3 Coleção Ictiológica, Instituto de Ciências e Tecnologia das Águas, Universidade Federal do Oeste do Pará, Santarém, Pará, Brazil; 4 Núcleo de Apoio a Pesquisa no Pará, Instituto Nacional de Pesquisas da Amazônia, Santarém, Pará, Brazil; University of Hyogo, JAPAN

## Abstract

The Amazon basin harbors a megadiverse fish fauna spread in an intricate network of big rivers and small streams. The Amazonian streams are home of many small sized fishes that remains poorly documented. In order to accelerate the scientific knowledge on these important aquatic systems we adopted a modern integrative approach joining morphology and molecular tools to investigate the ichthyofauna assemblages from low order streams situated on the lower Tapajós River Basin. *Cytochrome c Oxidase I* (COI) DNA barcodes from 252 specimens collected from 10 stream sites were obtained. The combined analysis revealed 29 species, 21 genera and 11 families. Cryptic diversity was evidenced in *Knodus* sp.1, *Aequidens epae* and *Copella callolepis*, in which deep genetic divergence were detected (intraspecific distances: 20.48%, 7.99% and 3.77%, respectively). The putative new species showed closer relationships with their counterparts occurring in the Tapajós-Xingu water drainages.

## Introduction

A significant part of the megadiverse Amazonian ichthyofauna inhabits in the extensive network of streams and small rivers (igarapés). These streams are scattered in all over the Amazonian uplands and lowlands territory, invading the forest matrix where they are mostly shadowed by forest canopy. The waters are usually acidic, with low temperature variation (24–26°C) and low primary productivity [1]. The riparian forest surrounding the watercourse plays pivotal ecosystem function as a nutrient source and the meanders, with a variety of obstacles in the channel (e.g. trunks, stones, and marginal vegetation roots); provide a succession of microhabitats ordinarily explored by tiny fish representative of orders Characiformes, Cichliformes, Siluriformes and Gymnotiformes [2], [3].

For a long time the ichthyofauna from Amazonian streams remained almost unknown and the major scientific efforts on the fish biodiversity, were concentrated around the easily accessible large floodplain rivers. In the last years several studies had been availed Amazonian streams exploring about sampling methods [4], [5], fish assemblages [6–9], fish ecology[7], [10] and environment degradation [11], [12].

The Amazonian rivers and streams have high endemism and taxonomic/ecological diversity, what could be appreciated by its about 2.500 fish species have already been described and more than 1.000 undesbribed species [13], [14]. In the present decade, hundreds of new fish species had been discovered in the Amazon region [14], [15], including in the streams of the lower Tapajós River [16], what is a warning that its real fish diversity continues severely underestimated. The Tapajós River drainage has a large fish diversity encompassing 494 recorded species, whose 17% are restricted from this water system [17] and 117 had been previously recorded in the streams from the lower portion of this basin [9].

Traditional morphology examinations remain as the favorite method for species identification, in use by many Brazilian fish scientists; however, complementary molecular studies (DNA barcoding) are promising and encouraged to accelerate the knowledge on fish taxonomy.

DNA barcoding [18] has been well established as a successful tool for species identification based on a short segment of mtDNA (*Cytochrome c Oxidase I* gene–COI). Many papers demonstrate the efficacy of DNA barcoding for recognizing and discover new fish species from the marine to freshwater habitats [19–24].

Studies of integrative taxonomy (morphology/DNA barcoding) really boosted the knowledge of ichthyofauna from important aquatic regions in Brazil. For instance, in the São Franscisco River [25], Paraíba do Sul River [26], Upper Paraná River [22], Mucuri River [23] and Jequitinhonha River [24]. Such approaches are still scarce for the ichythyofauna from Amazonian streams. The few contributions are recorded for groups delimited *a priori*, e.g. *Hyphessobrycon* [27], *Nannostomus* [28] and *Tetragonopterus* [29].

In the present study, we investigated the fish diversity from streams of the lower Tapajós River, by using morphology and molecular tools, in order to provide important taxonomic data to an underexplored and highly speciose Amazonian place. The necessity of taxonomic characterization and conservation policies to the Tapajós ichthyofauna arises from the imminent threats on the local aquatic biota regarding the impacts of gold mining industry and big hydroelectric power projects administered by government and multinational companies.

## Materials and methods

### Ethics statement

This research was conducted in accordance to the guidelines by the National Council for Control of Animal Experimentation and the Federal Board of Veterinary Medicine. The protocols were approved by the Committee on the Ethics of Animal Use of the Instituto Nacional de Pesquisas da Amazônia (040/2012; 041/2012). Sampled individuals were euthanized using a lethal concentration of Eugenol and whole specimens or piece of tissues were stored in ethanol. Fish sampling and tissue collect were authorized by the Brazilian Government and carried out under ICMBIO licenses (SISBIO 32653–3, 44215–1 and 44215–2).

### Study area

**Fig 1:**
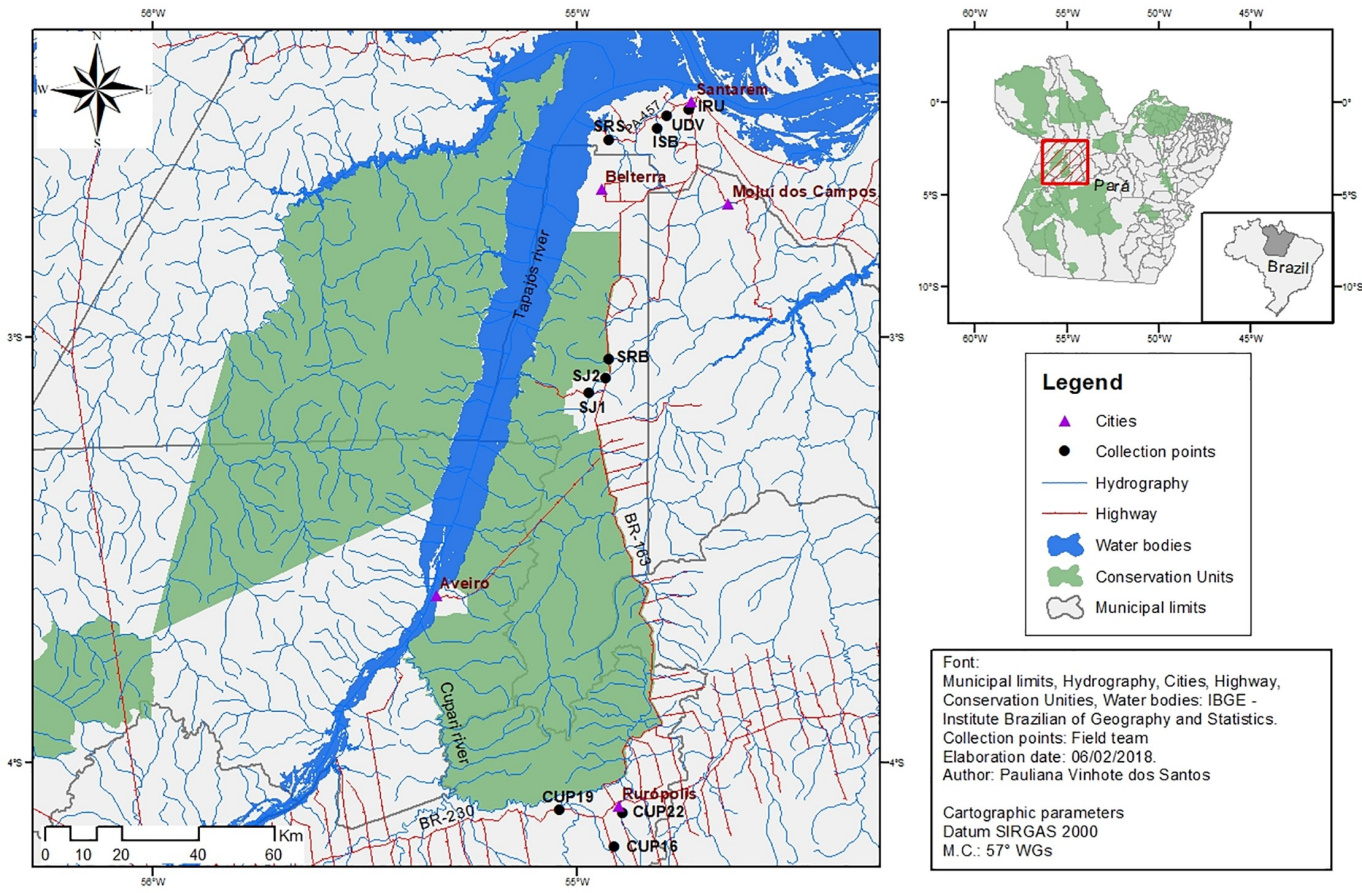
Map of collection sites of stream ichythyofauna at the region of lower Tapajós River.

**Fig 2:**
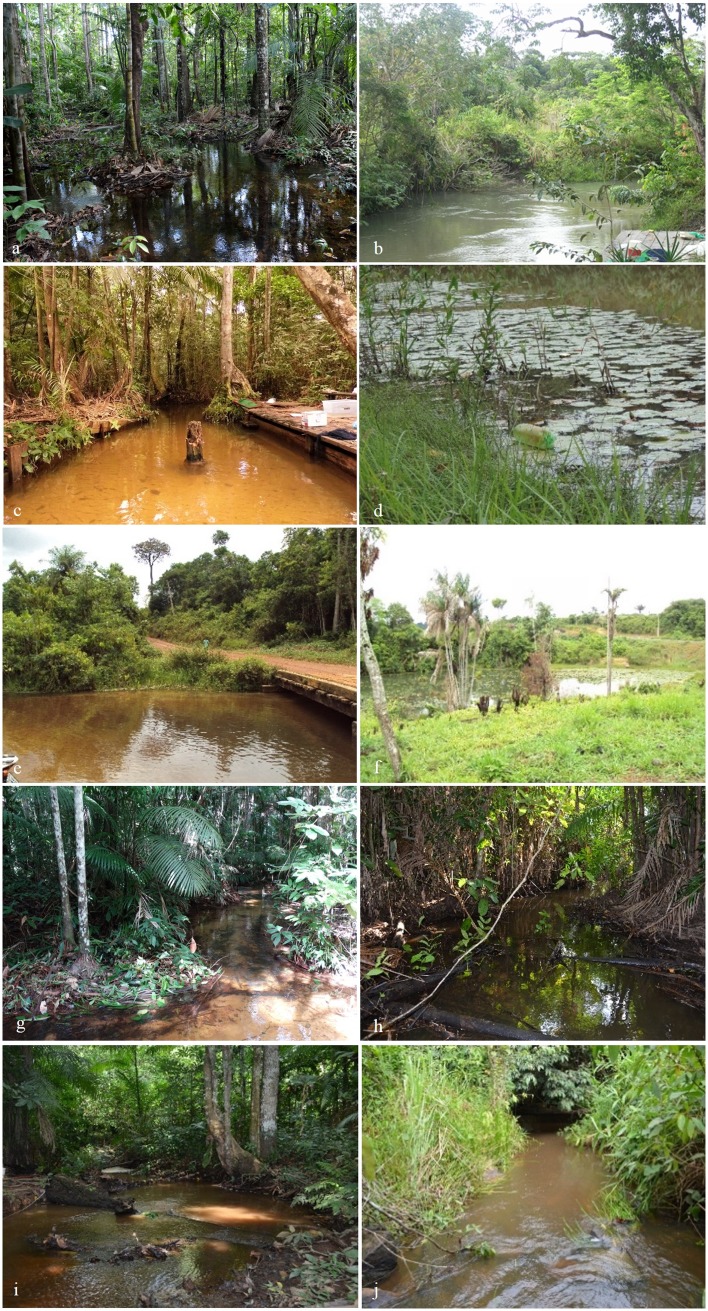
Stream sampled sites for fish assemblage study in the region of lower Tapajós River. a) União do Vegetal (UDV) stream; b) São Bras stream; c) Sonrisal stream; d) Irurá stream; e) São Jorge stream, site 1; f) São Jorge stream, site 2; g) Branco stream; h) unnamed strea at Cupari river (CUP 16); i) Guarú stream; j) Leitosinho stream.

A total of 10 low order streams, situated on the right bank of Tapajós River were surveyed. The streams are located in the municipality boundaries of Santarém, Belterra and Ruropólis, in Pará State, Brazil (Table 1, Figs 1 and 2).

**Table 1:** Geographic data of stream sites surveyed for fish assemblages at the region of lower Tapajós River.

Code	Stream name	Area	GPS coordinates	
			S	W
UDV	União do Vegetal	Santarém	-2.48100	-54.7890
ISB	São Bras		-2.51000	-54.8110
SRS	Sonrisal		-2.53500	-54.9240
IRU	Irurá		-2.46389	-54.7350
SJ1	São Jorge 1	Belterra	-3.13136	-54.9710
SJ2	São Jorge 2		-3.09447	-54.9309
BRC	Branco		-3.05100	-54.9250
CUP16	“unnamed stream”	Rurópolis	-4.19814	-54.9125
CUP19	Guarú		-4.10931	-55.0415
CUP22	Leitosinho		-4.11903	-54.8926

For convenience, the sampled sites were coded as follow: 1) União do Vegetal (UDV), 2) São Bras (ISB), 3) Igarapé Sonrisal (SRS), 4) Irurá (IRU), 5) São Jorge stream, site 1 (SJ1), 6) São Jorge stream, site 2 (SJ2), 7) Branco stream (BRC), 8) Unnamed stream at Cupari river (CUP16), 9) Guarú stream (CUP 19) and 10) Leitosinho stream (CUP 22).

#### Sampling and morphology analysis

Samples were collected from 2012 to 2015 (S1 File). Before each capture session, we delimited an aquatic parcel in a stretch of 50m long and blocked the stream channel in the extremity of the parcel using filament nets attached to the margins and the bottom of the stream [30]. The fishes were captured by three collectors using sieves and small nets (2 and 3m long) during 2 hours of active captures. Sample size for any given location ranged from 2 to 24 individuals. All the captured specimens were collected and when possible a maximum of 10 specimens per species and sampling event were chosen for DNA barcoding analysis, while the remaining ones were fixed and preserved for museum.

The collected fishes were provisionally classified in the field, as morphospecies. Then, each individual specimen was labelled and processed for photograph records and tissue sampling. Vouchers were fixed in formalin 10% for 24h, rinsed with water and moved to ethanol 70%, and deposited at Fish Collection of the Institute of Water Science and Technology, Federal University of Western Pará, Brazil (http://www.ufopa.edu.br/ufopa/institucional/unidades-academicas/icta/) (S2 File). The tissue collections were stored at Laboratory of Genetics and Biodiversity, Federal University of Western Pará, Brazil.

A definitive taxonomic identification at species level, based on the examination of morphologic characters and using identification keys [31–34] and taxonomic material deposited in scientific collections. If identification was not properly assigned to a specific species, “sp.”, “cf.” and “aff.” abreviations were applied [35].

#### Molecular methods

Before the specimen fixation, muscle tissue samples were extracted, preserved in Ethanol 96°GL and stored at -20°C. Genomic DNA was purified with the “salting out” protocol adapted by Vitorino and colleagues [36]. Briefly, the lysis step occurred in a microtube containing 440μL of lysis buffer (10mM Tris-HCl, 2mM EDTA, 400mM NaCl, 2% SDS) added with 10μL of proteinase K (10mg/mL), incubated in a water bath at 55°C by 3h or alternatively overnight. For DNA precipitation, we added 300μL of 5M NaCl and the microtubes were inverted manually and centrifuged by 10min at 10000 rpm. The DNA in the supernatant phase was collected and precipitated with 500μL of 100% isopropanol and centrifuged by 10min/10.000 rpm. The DNA was washed with 700μL of 70% ethanol, dried and reconstituted in 30 μL of sterile water. Finally, 5 μL of RNAse (10mg/mL) was added and incubated at 37°C by 30min. The purity and concentration of the extracted DNA were evaluated through electrophoresis with 1% agarose gel stained with Gelred (Biotium-Uniscience).

The DNA barcoding sequence from the 5’ region of the *Cytochrome c Oxidase I* (COI) mitochondrial gene was amplified by Polymerase Chain Reaction (PCR) using the universal primers FishF1–5’TCAACCAACCACAAAGACATTGGCAC3’ and Fish R1–5’TAGACTTCTGGGTGGCCAAAGAATCA3’ [19]. The reactions were performed in 25 μL final volume, containing 15 μL sterile H_2_O, 2.8 μL dNTP mix (1.25 mM), 2.5 μL buffer 10X (200 mM Tris-HCl (pH = 8,4) + 500 mM KCl), 2.5 μL de MgCl_2_ (50 mM), 0.5 μL of each primer (5μM), 0.2 μL Taq DNA polymerase (5U/μL) and 1 μL of genomic DNA. PCR conditions were as follows: 95°C (2min), 35 cycles of 94°C (30sec), 54°C (30sec) and 72°C (1min), followed by 72°C (10min). The reactions were performed in a Pxe 0.2 thermocycler (Thermo Scientific) and the PCR products were evaluated through electrophoresis with 1% agarose gel stained with Gelred (Biotium-Uniscience).

The PCR positive products were cleaned with columns system using the E.Z.N.A. Cycle Pure Kit (Omega Bio-tek) following the fabricant instructions. DNA barcoding sequences were obtained by di-desoxiterminal Sanger method using ABI PRISM Big Dye Terminator V.3 Cycle Sequencing kit (Applied Biosystems). Sequencing reactions were made in 96-well plates with final volume of 10μL, containing 5 μL of sterile H_2_O, 1.5 μL of sequencing buffer 5X, 0.5 μL of primer (10 μM), 1 μL of Big Dye mixture and 2 μL of PCR cleaned product. PCR conditions were as follows: 96°C (1 min); 35 cycles of 96°C (15 sec), 50°C (15 sec), and 60°C (4 min). The reactions were precipitated in ethanol/EDTA and dried at 90°C for 2min. The plates were resuspended with 10 μL Formamida Hi-Di, heated at 94°C for 3 min. and sequenced in ABI 3500 genetic analyser (Applied Biosystems).

#### Data analysis and species delimitation

DNA barcode sequences were previously edited to remove primer reads, to remove ambiguous bases, to inspect for premature stop codons and then be aligned with BioEdit [37] and MEGA v.7 [38]. The DNA barcode sequences and the standard associated metadata were uploaded to BOLD systems platform (www.boldsystems.org) and assigned to the “*Peixes de Igarapés da Bacia do Tapajós* (IGTAP label)” as part of campaign Br-BOL–Project 09. To analyse the barcode sequence database were used online BOLD tools: Distance Summary, Barcode Gap Analysis and Barcode Index Number System (BIN). To illustrate the phylogenetic arrangement of species and groups, we generated a dendrogram through Neighbor-Joining reconstruction under Kimura 2-parameters (K2P) model [39] using MEGA v.7 [38]. The statistical robustness of the branches was evaluated by bootstrap test with 1000 pseudo-replicates.

In order to delimit cryptic and candidate species we follow the criteria adopted in Pugedo and colleagues [24]. Potential candidate species were flagged if: 1) classified as Concordant BIN cluster; 2) present nearest neighbor distance (NND) higher than 2%. Cryptic species were recognized by possessing an intraspecific distance higher than 2% and no exihibit morphological distinctiveness between specimens *a priori*.

## Results

Based on the morphological assessment, the ichythyofaunal survey at streams from the lower Tapajós River revealed 29 species (spp.), 21 genera, 11 families (Table 2; S1 Fig). The Order Characiformes was the most abundant (17 spp., 58.6%) followed by Cichliformes (7 spp., 24.2%), Gymnotiformes (4 spp., 13.8%) and Siluriformes (1 spp., 3.4%). Ten taxa (OTU) posseses uncertainties in morphological species recognition and remained listed either as undescribed species (sp.) or with uncertain species-level identity (aff., cf., gr.).

**Table 2:** Species list and barcoded specimens of fishes sampled in streams from the region of lower Tapajós River. Taxa (OTU) with uncertainties in morphological species recognition are listed either as undescribed species (sp.) or with uncertain species-level identity (aff., cf., gr.).

ORDER											
Family	Collection sites										
*Species*	UDV	ISB	SRS	IRU	SJ 1	SJ 2	BRC	CUP 16	CUP 19	CUP 22	n
**CHARACIFORMES**											
**Characidae**											
*Bryconops* aff. *caudomaculatus* (Günther, 1864)					9	7					16
*Bryconops* cf. *transitoria* Steindachner, 1915		3		3							6
*Bryconops* aff. *melanurus* (Bloch, 1794)			8			10	1				19
*Hemigrammus* cf. *vorderwinkleri* Géry, 1963			5								5
*Hyphessobrycon agulha* Fowler, 1913		2									2
*Hyphessobrycon* gr. *heterorhabdus* (Ulrey, 1894)	15		4	5							24
*Hyphessobrycon heterorhabdus* (Ulrey, 1894)							9				9
*Hyphessobrycon ericae* Moreira & Lima, 2017					12	4	1				17
*Iguanodectes variatus* Géry, 1993	7		17								24
*Knodus* sp. 1									5	9	14
*Moenkhausia conspicua* Soares & Bührnheim, 2016					11						11
**Crenuchidae**											
*Characidium* cf. *zebra* Eigenmann, 1909									4		4
*Melanocharacidium* sp.										9	9
**Curimatidae**											
*Cyphocharax spiluropsis* (Eigenmann & Eigenmann,1889)					2						2
**Lebiasinidae**											
*Copella callolepis* (Regan, 1912)	14	1	1	2							18
**Serrasalmidae**											
*Myloplus rubripinnis* (Müller & Troschel, 1844)					1	4					5
**Erythrinidae**											
*Hoplias malabaricus* (Bloch, 1794)					1		6				7
**GYMNOTIFORMES**											
**Hypopomidae**											
*Microsternarchus bilineatus* Fernández-Yépez, 1968			3								3
**Rhamphichthyidae**											
*Gymnorhamphichthys petiti* Géry & Vu-Tân-Tuê, 1964							2				2
*Gymnorhamphichthys rondoni* (Ribeiro & Miranda, 1920)						2					2
**Gymnotidae**											
*Gymnotus coropinae* Hoedeman, 1962							2				2
**CICHLIFORMES**											
**Cichlidae**											
*Aequidens* sp.					2		1				3
*Aequidens epae* Kullander, 1995	1		2	1				4			8
*Apistogramma agassizii* (Steindachner, 1875)			2	3							5
*Apistogramma regani* Kullander, 1980					2						2
*Bujurquina* sp.										11	11
*Crenicichla semicincta* Steindachner, 1892					13						13
*Mesonauta festivus* (Heckel, 1840)				5							5
**SILURIFORMES**											
**Loricariidae**											
*Otocinclus vittatus* Regan, 1904		2									2
**TOTAL**	37	8	42	19	53	27	22	4	9	29	252

A total of 252 DNA barcode sequences longer than 500bp, without stop codons or indels, were yielded. The base composition showed a mean percentage of 18.06% (G), 27.76% (C), 23.49% (A) and 30.7% (T). The sample included from two to 24 individuals per species with an average of eight (Table 2). The mean intraspecific distance was 0.46% ranging from zero to 20.48% based on 1627 comparisons. This extraordinary high value of maximum intraspecific distance was recorded only in *Knodus* sp.1. The second higher value was observed in *Aequidens epae* (7.99%). The mean intrageneric distance was 14.79%, ranging from 3.55% to 22.14%, while within families the mean intrageneric distance was 24.25%, ranging from 4.12% to 32.76%.

The barcoding gap analysis revealed distances higher than 2% to the nearest neighbor (NND) for all the species (Table 3). Higher intraspecific divergence could be detected in *Knodus* sp. 1 (0 to 20.48%, with mean of 5.71%), additionally its maximum intraspecific distance overtake the nearest neighbor distance (*Otocinclus vittatus*, NND = 18.07%). Other three species presented maximum intraspecific distance above of the threshold of 2%, *Hoplias malabaricus* (2.48%), *Copella callolepis* (3.77%) and *Aequidens epae* (7.99%). The group named herein as *Knodus* sp.1 (n = 14) encompasses three lineages and four BINs (ACG7692, ACZ2936, ACZ2937 and ADC4164), with at least three species clearly evidenced through DNA barcodes. Additionally, *Copella callolepis* (n = 18) with three lineages and three BINs (ACX6532, ACH3210 and ACH3211) and *Aequidens epae* with two lineages and two BINs (ACH3650 and ADC3786) revealed cryptic diversity that were undetected with traditional morphology methods alone.

**Table 3:** BIN classification and measures of intraspecific genetic distances (I.D.) and nearest neighbor distances (NND) of fish species from the streams of the lower Tapajós River. Species with high intraspecific divergence (> 2%) were assigned in bold type. BIN classification follows—C (concordant), D (discordant) and S (singleton). Species complex were illuminated with blue shadow. The distances were estimated following Kimura-2-parameter model.

BIN (classification)	Morphological identification	Mean I.D.%	Max I.D.%	NND % (nearest species)
ACP6518(C)	*Bryconops* aff. *caudomaculatus*	0.15	0.51	4.48 (*B*. cf. *transitoria*)
ACH3629(C)	*Bryconops* aff. *melanurus*	0.34	0.28	17.53 (*B*. cf. *transitoria*)
ACG8555(D)	*Bryconops* cf. *transitoria*	0.06	0.17	4.48 (*B*. aff. *caudomaculatus*)
ADG9082(C)	*Hemigrammus* cf. *vorderwinkleri*	0.63	1.86	4.12 (*Hyphessobrycon ericae*)
ACH2868(C)	*Hyphessobrycon agulha*	0	0	14.22 (*Hyphessobrycon ericae*)
ACH2866(C)	*Hyphessobrycon* gr. *heterorhabdus*	0.17	0.67	9.15 (*H*. *heterorhabdus*)
ACH2867(C)	*Hyphessobrycon heterorhabdus*	0.11	0.31	9.15 (*H*. gr. *heterorhabdus*)
ACH3724(C)	*Hyphessobrycon ericae*	0.14	0.52	4.2 (*H*. cf. *vorderwinkleri*)
ACH3214(C)	*Iguanodectes variatus*	0.1	1.17	19.75 (*B*. aff. *melanurus*)
ACG7692(S), ACZ2936(C), ACZ2937(C), ADC4164(D)	***Knodus* sp.1**	**5.71**	**20.48**	18.07 (*Otocinclus vittatus*)
ACP6990(C)	*Moenkhausia conspicua*	0	0	20.28 (*Knodus* sp.1)
ACZ3230(C)	*Characidium* cf. *zebra*	0.19	0.38	18.87 (*Melanocharacidium* sp.)
ACZ3229(C)	*Melanocharacidium* sp.	0.18	0.57	18.87 (*C*. cf. *zebra*)
ACP7019(C)	*Cyphocharax spiluropsis*	0	0	18.32 (*H*. *malabaricus*)
ABZ3047(D)	***Hoplias malabaricus***	**0.7**	**2.48**	18.32 (*C*. *spiluropsis*)
ACX6532(C), ACH3210(C), ACH3211(S)	***Copella callolepis***	**0.99**	**3.77**	21.54 (*B*. cf. *transitoria*)
ACP6692(C)	*Myloplus rubripinnis*	0	0	19.36 (*Knodus* sp.1)
ACH3483(C)	*Aequidens* sp.	0.55	0.83	10.84 (*A*. *epae*)
ACH3650(C), ADC3786(C)	***Aequidens epae***	**2.97**	**7.99**	10.84 (*Aequidens* sp.)
AAJ1190(D)	*Apistogramma agasizii*	0.33	0.75	21.39 (*A*. *regani*)
ACP6660(C)	*Apistogramma regani*	0	0	21.39 (*A*. *agassizii*)
ACZ2664(C)	*Bujurquina* sp.	0	0	17.22 (*M*. *festivus*)
ACP7089(C)	*Crenicichla semicincta*	0.17	0.51	24.8 (*Aequidens* sp.)
AAX3846(C)	*Mesonauta festivus*	0.08	0.19	17.22 (*Bujurquina* sp.)
ACH2930(C)	*Gymnotus coropinae*	0	0	21.09 (*A*. *epae*)
ACH4111(C)	*Microsternachus bilineatus*	0	0	21.57 (*B*. aff. *caudomaculatus*)
ACH3829(C)	*Gymnorhamphichthys petiti*	1.09	1.09	5.55 (*G*. *rondoni*)
ACX7488(C)	*Gymnorhamphichthys rondoni*	0.17	0.17	5.55 (*G*. *petiti*)
ACH2526(C)	*Otocinclus vittatus*	0.16	0.16	18.07 (*Knodus* sp. 1)

**Fig 3:**
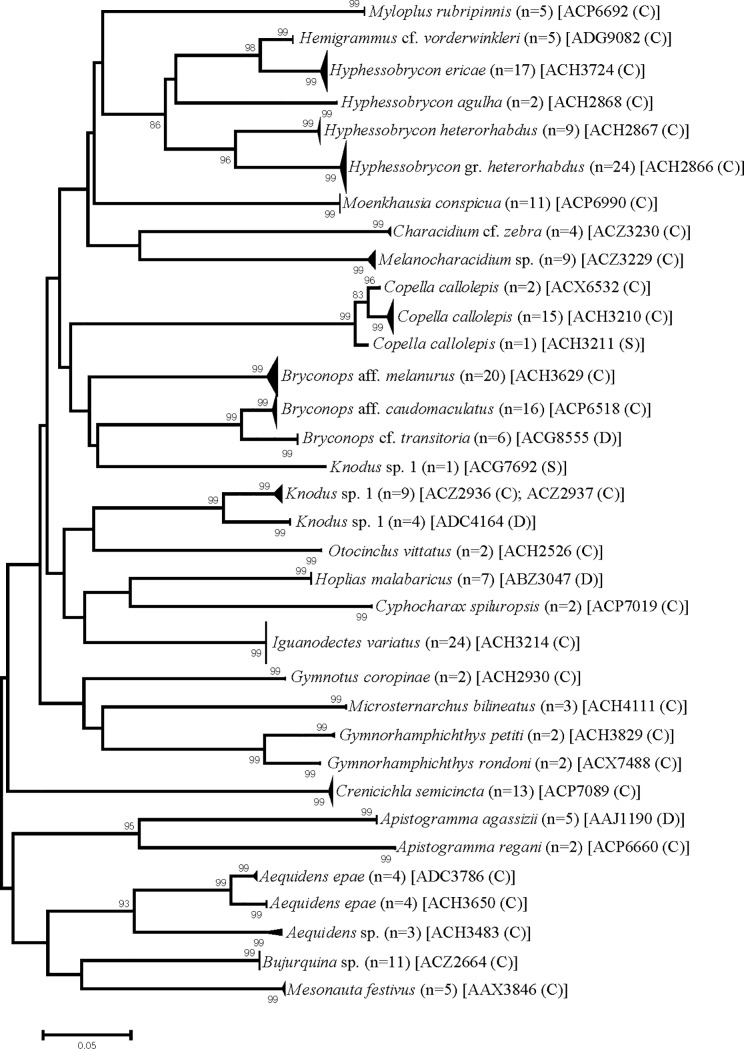
Neighbor-joining phylogenetic reconstruction based on DNA barcoding sequences of fish groups from streams of the lower Tapajós River. The values on the branches are measures of Bootstrap with 1000 pseudoreplicates. The matching between morphological and molecular species identification were assigned by BIN categories: C (concordant), D (discordant), S (Singleton).

The clusters recovered from the barcodes phylogenetic reconstruction indicated fully agreement to the species assigned a priori by morphological based identification (Fig 3). With the exception of *Knodus* sp.1 that was splited into multiple branches as a paraphyletic array (Fig 3). On the other hand, a Barcode Index Number (BIN) analysis, implemented with the Boldsystems workbench, revealed 35 clusters which 29 were classified as concordant (clusters constituted of one species); two clusters are singletons (*Knodus* sp. 1 –IGTAP264-16, BIN-ACG7692 and *Copella callolepis*–IGTAP045-13, BIN-ACH3211) and finally four clusters discordant (clusters constituted of more than one species). The latter included the following species: *Hoplias malabaricus* (BIN-ABZ3047), *Knodus* sp. 1 (BIN-ADC4164), *Bryconops* cf. *transitoria* (BIN-ACG8555) and *Apistogramma agassizii* (BIN-AAJ1190).

The integrative approach following Pugedo et al. (2016) criteria for species delimitation highlighted 25 species (BIN concordant and NND > 2%), Table 3. Based on morphology traits examination we identified fifteen of these species, but the remaining 10 species did not have their taxonomic status precisely determined. Some of them presumably have poor diagnose characters resulting taxonomic confusion with congeners (e.g. *Bryconops* aff. *caudomaculatus*, *Hemigrammus* cf. *vorderwinkleri*), personal observation (FRVR). However, other taxa were better characterized as undescribed new species candidates (e.g. *Hyphessobrycron* gr. *heterorhabdus*, *Melanocharacidium* sp., *Aequidens* sp. and *Bujurquina* sp.).

## Discussion

The Brazilian inland aquatic biota has been investigated for centuries, but we are far from to consider this megadiverse group reasonably well studied. A good example to highlight our ignorance on this theme could be the recently amazing discovery of a new fish family (Tarumaniidae–Characiformes) from deep fossorial Amazonian habitats [40].

Based on an integrative approach we delimited 29 nominal fish species from Amazonian streams and some of them clearly harbour cryptic diversity. The DNA barcoding evidence suggests that the most promising taxa with putative new undescribed species are *Knodus* sp.1, *Aequidens epae* and *Copella callolepis*. Three species (*Bryconops* cf. *transitoria*, *Hoplias malabaricus* and *Apistogramma agasizii*) were recognized based on morphology, but not with the present adopted DNA barcode criteria, since its BIN resulted discordant. Such discordance of BIN result can emerge from erroneous entries stored in BOLDsystems databases and comparisons with known complex of undescribed species (e.g. *Hoplias malabaricus*).

The genus *Knodus* Eigenmann [41] is a disputed taxon that has been classified as *Bryconamericus* synonym, e.g. [42–45]. Ferreira [46] considers *Knodus* as a valid monophyletic genus including four *Bryconamericus* species. Recently, new species have been described in *Knodus*, which currently encompasses 29 nominal species [47].

Thomaz and colleagues [48] based on a molecular phylogeny supports the paraphyletic status for *Knodus*-*Bryconamericus* and observed that *Knodus* species aligned with other taxa are splited into two clades with Amazon-Orinoco distribution. The first clade was designated as *Knodus sensu stricto* and includes the *Knodus* species type (*K*. *meridae*) plus 13 species of this genus, 11 *Bryconamericus* species and *Bryconadenus tanaothorus*. Our *Knodus* sp. 1 specimens were collected from the Cupari-Tapajós drainage. In the Tapajós Basin have been previously recorded *K*. *dorsomaculatus* [49], *K*. cf. *chapadae*, *Knodus* sp. Tapajós, *Knodus* sp. Teles Pires [48], *K*. *heteresthes*, *K*. *shinahota* and *Knodus* sp. [9].

**Fig 4:**
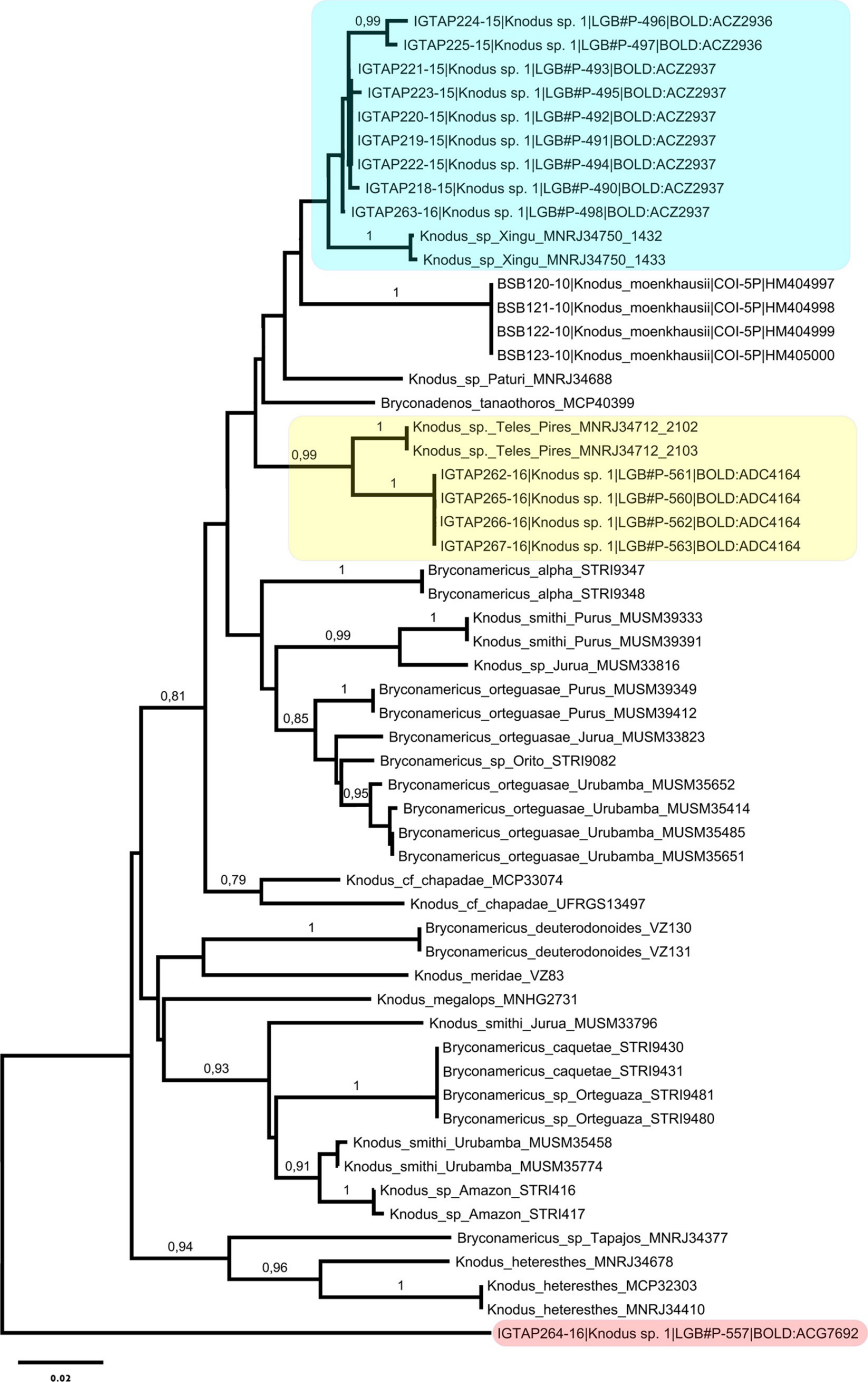
Neighbor-Joining phylogenetic tree of *Knodus sensu stricto* showing the association of the *Knodus* sp.1 putative new species (BINs ACZ2936, ACZ2937, ADC4164) with *Knodus* sp. from Xingu and Teles Pires Rivers, the clades were shadowed with blue and yellow colors. The *Knodus* sp. 1 (BIN ACG7692), shadowed with red color, was evidenced as a distant lineage apart from *Knodus sensu stricto*. The values in the branches are bootstrap measures of 1000 pseudoreplicates.

In order to explore the phylogenetic relationships of *Knodus* sp.1 from the Cupari-Tapajós drainage we assembled DNA barcodes downloaded from the GenBank (accessions: KF210030 –KF210276), assigned to the species included in the *Knodus sensu stricto* [48]. The putative species *Knodus* sp.1 BINs (ACZ2936; ACZ2937) showed a closer relation to *Knodus* sp. Xingu, whereas the BIN (ADC4164) was sister aligned to *Knodus* sp. Teles Pires. The *Knodus* sp.1 singleton BIN (ACG7692) branched as a separated lineage clearly distinct from the all species included within *Knodus sensu stricto* (Fig 4). Therefore, this complementary analysis revealed that *Knodus* sp.1 hides three new species that belong to *Knodus sensu stricto* [48] and carry molecular and geographic affinities with taxa from the Xingu-Tapajós drainages. On the other hand, the BIN ACG7692 should not be *Knodus* taxa. A comparison with other Stevardiinae (S2 Fig) suggest that it is related to the genus *Tyttocharax* (Xenurobriconini).

The genus *Aequidens* Eigenmann and Bray [50] encompasses 18 valid species, which are largely distributed along the South America drainages [47], [51], [52]. Previous records of *Aequidens* on the Tapajós basin were pointed to *A*. *epae*, *A*. *mauesanus* and *A*. *tetramerus* [9], [53]. Additionally, Silva-Oliveira and colleagues [9] reports on *Aequidens* sp. from the Cupari River drainage. In the present study, we found *A*. *epae* splited into two lineages that clearly diverged as full species (BINs ACH3650 and ADC3786). The first clade occurred in lowest portion of the Tapajós River near the confluence with the Amazonas River, instead of the later that occurred at the Cupari River drainage. A phylogenetic analysis of *Aequidens* from Tapajós, supplemented with DNA barcodes of congeners downloaded from Boldsystems, revealed that *A*. *epae* Cupari River nested with *A*. *diadema* (GenBank accession: GU817291) while *A*. *epae* Lowest Tapajós River was linked to this branch as a basal lineage. On the other hand, our specimens delimited as *Aequidens* sp. from São Jorge/Branco streams (BIN ACH3483) showed phylogenetic affinity with *A*. *tetramerus*. In summary, it is reasonable to point at least three putative new *Aequidens* species from the Tapajós basin: *Aequidens* sp. Cupari River [9], *A*. *epae* Cupari River (BIN ADC3786) and *Aequidens* sp. São Jorge/Branco streams.

The lebiasinid genus *Copella* was recently revised and encompasses 6 nominal valid species: *C*. *arnoldi*, *C*. *callolepis*, *C*. *compta*, *C*. *eigenmanni*, *C*. *nattereri*, and *C*. *vilmae* [54]. Based on morphological traits all the specimens recovered from the Tapajós basin streams (present study) were identified as *C*. *callolepis*, however, the molecular evidences suggest a species complex.

Two of the putative new species (*C*. *callolepis*—BIN ACH3211, BIN ACX6532) occurred at UDV and Irurá streams, both places situated in the periurban area of Santarém, the most populous city in the lower Tapajós region. The third species (BIN ACH3210) occurred at UDV, São Bras and Sonrisal streams. These aquatic systems are situated near of an important paved road (PA-457) that links Santarém to Alter do Chão Village. Because of their vicinity with an urban centre, there are high disturbance in the natural habitats associated with distinct human pressures as marginal deforestation, aquatic pollution, water collect for domestic and aquaculture usage. This scenario is threatening for long-term persistence of such populations and conservation/management plans, as well as further studies on integrative basis should be carried out, in order to minimize the associated risk of premature local extinctions of new undescribed fish species.

DNA barcode studies in 14 nominal *Nannostomus* species (a lebiasinid close to *Copella*) have detected high genetic divergence (>2%) within a single putative taxonomic entity. Four species presented deep lineage divergences. These divergences were evident between individuals taxonomically identiied as *N*. *digrammus* (9.80% ± 1.0%), *N*. *trifasciatus* (8.1% ± 0.7%), *N*. *unifasciatus* (7.1% ± 0.7%), and *N*. *eques* (4.0% ± 0.4%). For *N*. *digrammus* and *N*. *trifasciatus*, in particular, the estimated divergences in some lineages were so high that doubt about their conspecific status is raised, suggesting the existence of hidden diversity [28].

DNA barcodes has been largely assumed as a powerful tool for species delimitation and has been effective to discover new fish species; however, such operators enrolled in this methodology may contribute to diminish its precision and resolution power. For instance, in the present study our DNA barcode approach found 25 species based on Pugedo’s criteria while the morphology examination pointed 29 species. These discrepancies arose because few OTU did not match with molecular and morphology identifications, since them were classified as discordant BINs or species complex. Factors that contribute to these inconsistencies could be the deficient library of standard DNA barcodes released from BOLDSystems platform and the occurrence of records with imprecise or erroneous entries uploaded in this repository, and that are being used for BIN analysis.

The insufficient coverage of the public repositories for standard DNA barcode sequences of Neotropical freshwater fishes is well demonstrated when 67% of the species listed in the present paper did not have any DNA barcode previously published. Moreover, DNA barcodes failed to delimit *Bryconops* cf. *transitoria* (BIN ACG8555) despite a consistent cluster of six individuals with mean intraspecific distance of 0.06 and NN distance of > than 2%. This BIN resulted discordant due to a clustering of *B*. cf. *transitoria* with *Hyphessobrycon pulchripinnis* previously deposited in BOLDsystems from a unique individual [27]. How these species are clearly distinguished by morphology, we suspected that it is a case of an erroneous entry of *H*. *pulchripinnis*. Surprisingly, the NN distance of *H*. *pulchripinnis* and *H*. *rosaceus* was 22.55, two times bigger than the second NN distance pair, 10.45 between *H*. *eques* and *H*. *copelandi* [27].

The Tapajós basin is a hotspot for stream ichythyofauna and the integrative taxonomy is a powerful methodology for prospecting biodiversity in the Amazonian waters. Further studies are advised to bring light on the obscure taxonomy of *Knodus* likewise on the phylogenetic and geographic relationships of the regional stream fish assemblages.

## Supporting information

S1 File Specimen data DNA barcoding IGTAP from BOLD. (XLS)

S2 File Specimens examined (barcoded). (PDF)

S1 Fig Photograph records (species examined). (TIF)

S2 Fig Phylogenetic relations in Stevardini showing the clade *Knodus* sensu stricto and the closer affinity between *Tyttocharax tambopatensis* and *Knodus* sp.1 (IGTAP 264–16). (TIF)
