# Supplementary material for: DNA barcoding of fish fauna from low order streams of Tapajós River basin
Source: PLoS One. 2018 Dec 21;13(12):e0209430. doi: 10.1371/journal.pone.0209430 (PMC6303048; doi:10.1371/journal.pone.0209430)
Supplement: S2 File — (PDF) [file pone.0209430.s002.pdf]

CHARACIFORMES: *Bryconops* aff. *caudomaculatus* (IGTAP152-15, KAL2 MF2-1, SJ2; IGTAP154-15, KAL2 MF2-3, SJ2; IGTAP082-14, KAL1 MF2-1, SJ1; IGTAP172-15, KAL1 MF12-6, SJ1; IGTAP171-15, KAL1 MF12-5, SJ1; IGTAP170-15, KAL1 MF12-3, SJ1; IGTAP160-15, KAL1 MF2-10, SJ1; IGTAP159-15, KAL1 MF2-9, SJ1; IGTAP085-14, KAL1 MF2-7, SJ1; IGTAP156-15, KAL2 MF2-6, SJ2; IGTAP157-15, KAL2 MF2-7, SJ2; IGTAP153-15, KAL2 MF2-2, SJ2; IGTAP083-14, KAL1 MF2-3, SJ1; IGTAP084-14, KAL1 MF2-4, SJ1; IGTAP162-15, KAL2 MF2-10, SJ2; IGTAP155-15, KAL2 MF2-5, SJ2). *Bryconops* cf. *transitoria* (IGTAP165-15, IRU MF22, IRU; IGTAP023-13, Msp3-14, ISB; IGTAP164-15, IRU MF21, IRU; IGTAP024-13, Msp3-15, ISB; IGTAP025-13, Msp3-13, ISB; IGTAP166-15, IRU MF23, IRU). *Bryconops* aff. *melanurus* (IGTAP022-13, Msp3-9, SRS; IGTAP021-13, Msp3-3, SRS; IGTAP020-13, Msp3-1, SRS; IGTAP019-13, Msp3-8, SRS; IGTAP018-13, Msp3-7, SRS; IGTAP017-13, Msp3-4, SRS; IGTAP016-13, Msp3-2, SRS; IGTAP015-13, Msp3-11, SRS; IGTAP118-14, KAL2 MF12-9, SJ2; IGTAP168-15, KAL2 MF12-7, SJ2; IGTAP169-15, KAL2 MF12-8, SJ2; IGTAP112-14, KAL2 MF12-1, SJ2; IGTAP119-14, KAL2 MF12-10, SJ2; IGTAP113-14, KAL2 MF12-2, SJ2; IGTAP114-14, KAL2 MF12-3, SJ2; IGTAP115-14, KAL2 MF12-4, SJ2; IGTAP116-14, KAL2 MF12-5, SJ2; IGTAP117-14, KAL2 MF12-6, SJ2; IGTAP026-13, Msp3-12, IGB). *Hemigrammus* cf. *vorderwinkleri* (IGTAP311-17, KAL3MF24-6, SRS; IGTAP308-17, KAL3 MF24-8, SRS; IGTAP303-17, KAL3 MF24-4, SRS; IGTAP302-17, KAL3 MF24-3, SRS; IGTAP310-17, KAL3 MF24-7, SRS). *Hyphessobrycon agulha* (IGTAP031-13, Msp4-26, ISB; IGTAP032-13, Msp4-27, ISB). *Hyphessobrycon* gr. *heterorhabdus* (IGTAP027-13, Msp4-1, SRS; IGTAP125-15, KAL4 MF28-1, UDV; IGTAP039-13, Msp4-20, UDV; IGTAP038-13, Msp4-9, SRS; IGTAP036-13, Msp4-7, SRS; IGTAP034-13, Msp4-2, SRS; IGTAP030-13, Msp4-17, UDV; IGTAP142-15, IRU MF28-9, IRU; IGTAP141-15, IRU MF28-6, IRU; IGTAP140-15, IRU MF28-5, IRU; IGTAP139-15, IRU MF28-4, IRU; IGTAP138-15, IRU MF28-1, IRU; IGTAP137-15, KAL4 MF28-16, UDV; IGTAP136-15, KAL4 MF28-12, UDV; IGTAP135-15, KAL4 MF28-11, UDV; IGTAP134-15, KAL4 MF28-10, UDV; IGTAP133-15, KAL4 MF28-9, UDV; IGTAP132-15, KAL4 MF28-8, UDV; IGTAP131-15, KAL4 MF28-7, UDV; IGTAP130-15, KAL4 MF28-6, UDV; IGTAP129-15, KAL4 MF28-5, UDV; IGTAP128-15, KAL4 MF28-4, UDV; IGTAP127-15, KAL4 MF28-3, UDV; IGTAP126-15, KAL4 MF28-2, UDV). *Hyphessobrycon heterorhabdus* (IGTAP037-13, Msp4-36, BRC; IGTAP028-13, Msp4-34, BRC; IGTAP029-13, Msp4-40, BRC; IGTAP033-13, Msp4-39, BRC; IGTAP035-13, Msp4-32, BRC; IGTAP040-13, Msp4-33, BRC; IGTAP041-13, Msp4-35, BRC; IGTAP042-13, Msp4-41, BRC; IGTAP043-13, Msp4-42, BRC). *Hyphessobrycon ericae* (IGTAP143-15, KAL2 MF1-2, SJ2; IGTAP144-15, KAL2 MF1-3, SJ2; IGTAP145-15, KAL2 MF1-6, SJ2; IGTAP076-14, KAL1 MF1-7, SJ1; IGTAP073-14, KAL1 MF1-4, SJ1; IGTAP072-14, KAL1 MF1-3, SJ1; IGTAP081-14, KAL1 MF1-12, SJ1; IGTAP080-14, KAL1 MF1-11, SJ1; IGTAP079-14, KAL1 MF1-10, SJ1; IGTAP078-14, KAL1 MF1-9, SJ1; IGTAP146-15, KAL2 MF1-8, SJ2; IGTAP075-14, KAL1 MF1-6, SJ1; IGTAP071-14, KAL1 MF1-2, SJ1; IGTAP074-14, KAL1 MF1-5, SJ1; IGTAP070-14, KAL1 MF1-1, SJ1; IGTAP077-14, KAL1 MF1-8, SJ1; IGTAP060-13, Msp27-2, BRC). *Iguanodectes variatus* (IGTAP012-13, Msp1-2, SRS; IGTAP013-13, Msp1-4, SRS; IGTAP014-13, Msp1-12, UDV; IGTAP006-13, Msp1-19, UDV; IGTAP007-13, Msp1-3, SRS; IGTAP005-13, Msp1-14, UDV; IGTAP004-13, Msp1-9, SRS; IGTAP003-13, Msp1-8, SRS; IGTAP002-13, Msp1-15, UDV; IGTAP184-15, KAL3 MF27-7, SRS; IGTAP183-15,

KAL3 MF27-5, SRS; IGTAP182-15, KAL3 MF27-3, SRS; IGTAP181-15, KAL3 MF27-2, SRS; IGTAP180-15, KAL3 MF27-1, SRS; IGTAP187-15, KAL3 MF27-10, SRS; IGTAP001-13, Msp1-5, SRS; IGTAP186-15, KAL3 MF27-9, SRS; IGTAP188-15, KAL3 MF27-11, SRS; IGTAP185-15, KAL3 MF27-8, SRS; IGTAP189-15, KAL4 MF27-1, UDV; IGTAP008-13, Msp1-10, SRS; IGTAP009-13, Msp1-11, SRS; IGTAP010-13, Msp1-13, UDV; IGTAP011-13, Msp1-18, UDV). *Knodus* sp. 1 (IGTAP225-15, Pt22Sp1-8, CUP22; IGTAP218-15, Pt22Sp1-1, CUP22; IGTAP219-15, Pt22Sp1-2, CUP22; IGTAP220-15, Pt22Sp1-3, CUP22; IGTAP221-15, Pt22Sp1-4, CUP22; IGTAP222-15, Pt22Sp1-5, CUP22; IGTAP223-15, Pt22Sp1-6, CUP22; IGTAP224-15, Pt22Sp1-7, CUP22; IGTAP262-16, Pt19Sp1-26, CUP19; IGTAP263-16, Pt22Sp1-9, CUP22; IGTAP265-16, Pt19Sp1-25, CUP19; IGTAP266-16, Pt19Sp1-27, CUP19; IGTAP267-16, Pt19Sp1-28, CUP19; IGTAP264-16, Pt19Sp1-22, CUP19). *Moenkhausia conspicua* (IGTAP090-14, KAL1 MF3-7, SJ1; IGTAP091-14, KAL1 MF3-8, SJ1; IGTAP092-14, KAL1 MF3-9, SJ1; IGTAP093-14, KAL1 MF3-10, SJ1; IGTAP094-14, KAL1 MF3-11, SJ1; IGTAP095-14, KAL1 MF3-12, SJ1; IGTAP096-14, KAL1 MF3-13, SJ1; IGTAP087-14, KAL1 MF3-4, SJ1; IGTAP086-14, KAL1 MF3-3, SJ1; IGTAP088-14, KAL1 MF3-5, SJ1; IGTAP089-14, KAL1 MF3-6, SJ1). *Characidium* cf. *zebra* (IGTAP271-16, Pt19Sp4-14, CUP19; IGTAP246-15, Pt19Sp4-22, CUP19; IGTAP245-15, Pt19Sp4-21, CUP19; IGTAP272-16, Pt19Sp4-19, CUP19). *Melanocharacidium* sp. (IGTAP230-15, Pt22Sp4-5, CUP22; IGTAP234-15, Pt22Sp4-10, CUP22; IGTAP233-15, Pt22Sp4-9, CUP22; IGTAP270-16, Pt22Sp4-7, CUP22; IGTAP269-16, Pt22Sp4-6, CUP22; IGTAP228-15, Pt22Sp4-2, CUP22; IGTAP227-15, Pt22Sp4-1, CUP22; IGTAP232-15, Pt22Sp4-8, CUP22; IGTAP229-15, Pt22Sp4-3, CUP22). *Cyphocharax spiluropsis* (IGTAP100-14, KAL1 MF5-1, SJ1; IGTAP101-14, KAL1 MF5-2, SJ1). *Copella callolepis* (IGTAP046-13, Msp5-27, ISB; IGTAP287-16, KAL4 MF21-13, UDV; IGTAP286-16, KAL4 MF21-10, UDV; IGTAP285-16, KAL4 MF21-9, UDV; IGTAP284-16, KAL4 MF21-7, UDV; IGTAP283-16, KAL4 MF21-6, UDV; IGTAP282-16, KAL4 MF21-5, UDV; IGTAP281-16, KAL4 MF21-4, UDV; IGTAP280-16, KAL4 MF21-3, UDV; IGTAP279-16, KAL4 MF21-2, UDV; IGTAP278-16, KAL4 MF21-1, UDV; IGTAP175-15, IRU MF21-1, IRU; IGTAP176-15, IRU MF21-3, IRU; IGTAP177-15, KAL4 MF21-8, UDV; IGTAP048-13, Msp5-14, SRS; IGTAP047-13, Msp5-15, UDV; IGTAP045-13, Msp5-13, UDV; IGTAP044-13, Msp5-20, UDV). *Myloplus rubripinnis* (IGTAP097-14, KAL1 MF4-1, SJ1; IGTAP121-14, KAL2 MF20-2, SJ2; IGTAP122-14, KAL2 MF20-3, SJ2; IGTAP098-14, KAL2 MF4-5, SJ2; IGTAP099-14, KAL2 MF4-6, SJ2). *Hoplias malabaricus* (IGTAP301-17, KAL1 MF11-1, SJ1; IGTAP069-13, IGB-6, BRC; IGTAP064-13, IGB-1, BRC; IGTAP065-13, IGB-2, BRC; IGTAP067-13, IGB-4, BRC; IGTAP066-13, IGB-3, BRC; IGTAP068-13, IGB-5, BRC). **GYMNOTIFORMES:** *Microsternarchus bilineatus* (IGTAP052-13, Msp8-4, SRS; IGTAP051-13, Msp8-3, SRS; IGTAP053-13, Msp8-2, SRS). *Gymnorhamphichthys petiti* (IGTAP063-13, Msp15-1, BRC; IGTAP307-17, IRU MF13-1, IRU). *Gymnorhamphichthys rondoni* (IGTAP174-15, KAL2 MF13-2, SJ2; IGTAP173-15, KAL2 MF13-1, SJ2). *Gymnotus coropinae* (IGTAP061-13, Msp30-2, BRC; IGTAP062-13, Msp30-1, BRC). **CICHLIFORMES:** *Aequidens* sp. (IGTAP055-13, Msp10-20, BRC; IGTAP151-15, KAL1 MF8-2, SJ1; IGTAP150-15, KAL1 MF8-1, SJ1). *Aequidens epae* (IGTAP201-15, IRU MF7-3, IRU; IGTAP054-13, Msp10-11, UDV; IGTAP056-13, Msp10-3, SRS; IGTAP057-13, Msp10-4, SRS; IGTAP261-16, Pt16Sp7-9, CUP16; IGTAP258-16, Pt16Sp7-1, CUP16; IGTAP259-16, Pt16Sp7-2,

CUP16; IGTAP260-16, Pt16Sp7-6, CUP16). *Apistogramma agassizii* (IGTAP179-15, IRU MF22-2, IRU; IGTAP178-15, IRU MF22-1, IRU; IGTAP268-16, IRU MF22-4, IRU; IGTAP050-13, Msp6-10, SRS; IGTAP049-13, Msp6-9, SRS). *Apistogramma regani* (IGTAP110-14, KAL1 MF7-1, SJ1; IGTAP111-14, KAL1 MF7-2, SJ1). *Bujurquina* sp. (IGTAP275-16, Pt22Sp11-6, CUP22; IGTAP273-16, Pt22Sp11-1, CUP22; IGTAP274-16, Pt22Sp11-2, CUP22; IGTAP277-16, Pt22Sp11-10, CUP22; IGTAP236-15, Pt22Sp11-4, CUP22; IGTAP240-15, Pt22Sp11-8, CUP22; IGTAP237-15, Pt22Sp11-5, CUP22; IGTAP241-15, Pt22Sp11-9, CUP22; IGTAP235-15, Pt22Sp11-3, CUP22; IGTAP247-15, Pt18Sp29-9, CUP22; IGTAP276-16, Pt22Sp11-7, CUP22). *Crenicichla semicincta* (IGTAP149-15, KAL1 MF6-2, SJ1; IGTAP105-14, KAL1 MF6-8, SJ1; IGTAP167-15, KAL1 MF31-3, SJ1; IGTAP106-14, KAL1 MF6-10, SJ1; IGTAP102-14, KAL1 MF6-3, SJ1; IGTAP103-14, KAL1 MF6-4, SJ1; IGTAP104-14, KAL1 MF6-6, SJ1; IGTAP148-15, KAL1 MF6-1, SJ1; IGTAP124-14, KAL1 MF31-2, SJ1; IGTAP109-14, KAL1 MF6-15, SJ1; IGTAP123-14, KAL1 MF31-1, SJ1; IGTAP108-14, KAL1 MF6-14, SJ1; IGTAP107-14, KAL1 MF6-11, SJ1). *Mesonauta festivus* (IGTAP212-15, IRU MF35-9, IRU; IGTAP210-15, IRU MF35-5, IRU; IGTAP211-15, IRU MF35-6, IRU; IGTAP207-15, IRU MF35-1, IRU; IGTAP208-15, IRU MF35-2, IRU). **SILURIFORMES:** *Otocinclus vittatus* (IGTAP058-13, Msp16-11, ISB; IGTAP059-13, Msp16-6, ISB).
