# Supplementary figures and images for: DNA barcoding of fish fauna from low order streams of Tapajós River basin
Source: PLoS One. 2018 Dec 21;13(12):e0209430. doi: 10.1371/journal.pone.0209430 (PMC6303048; doi:10.1371/journal.pone.0209430)

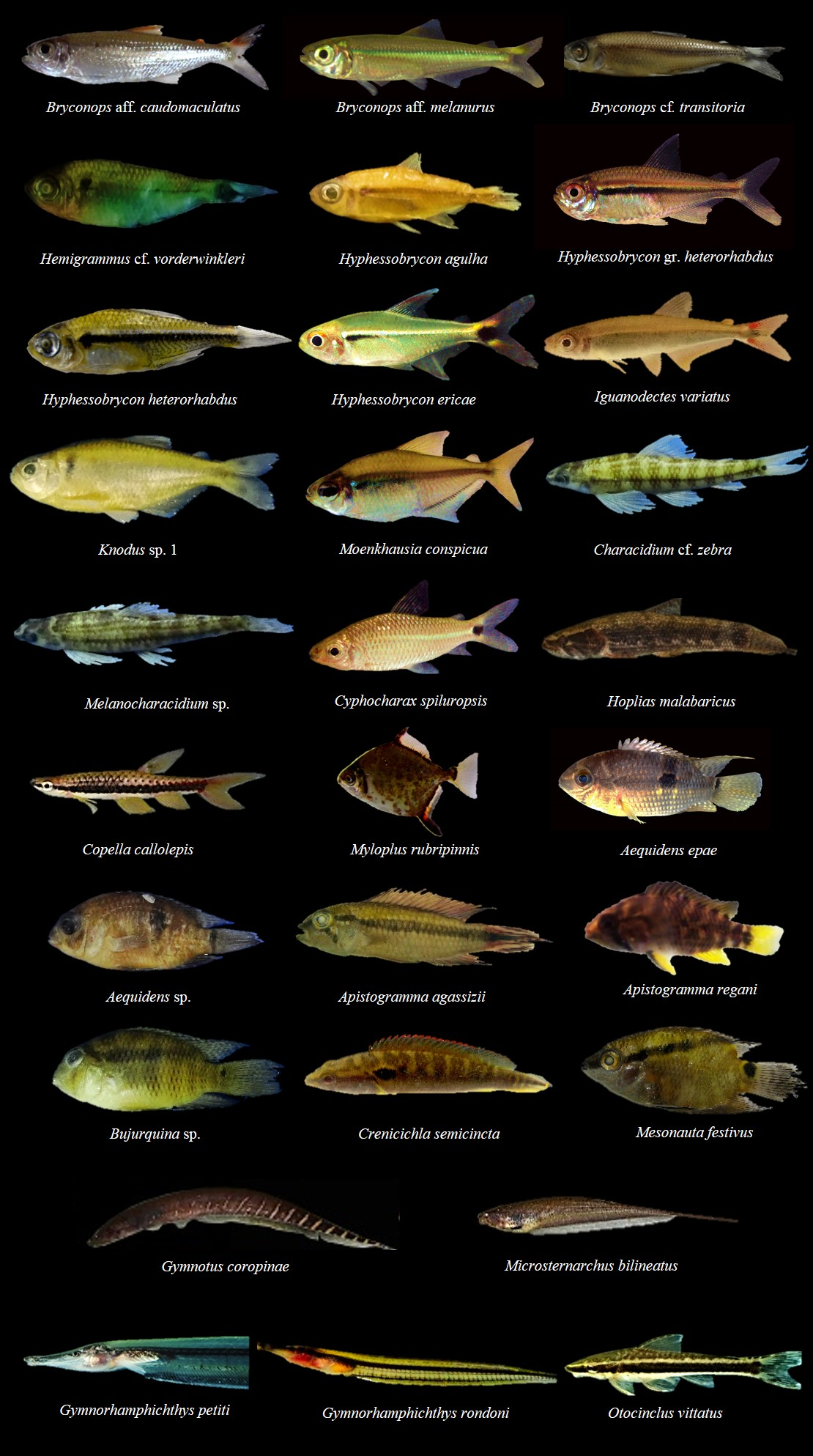

Supplement: S1 Fig — (TIF) [file pone.0209430.s003.tif]

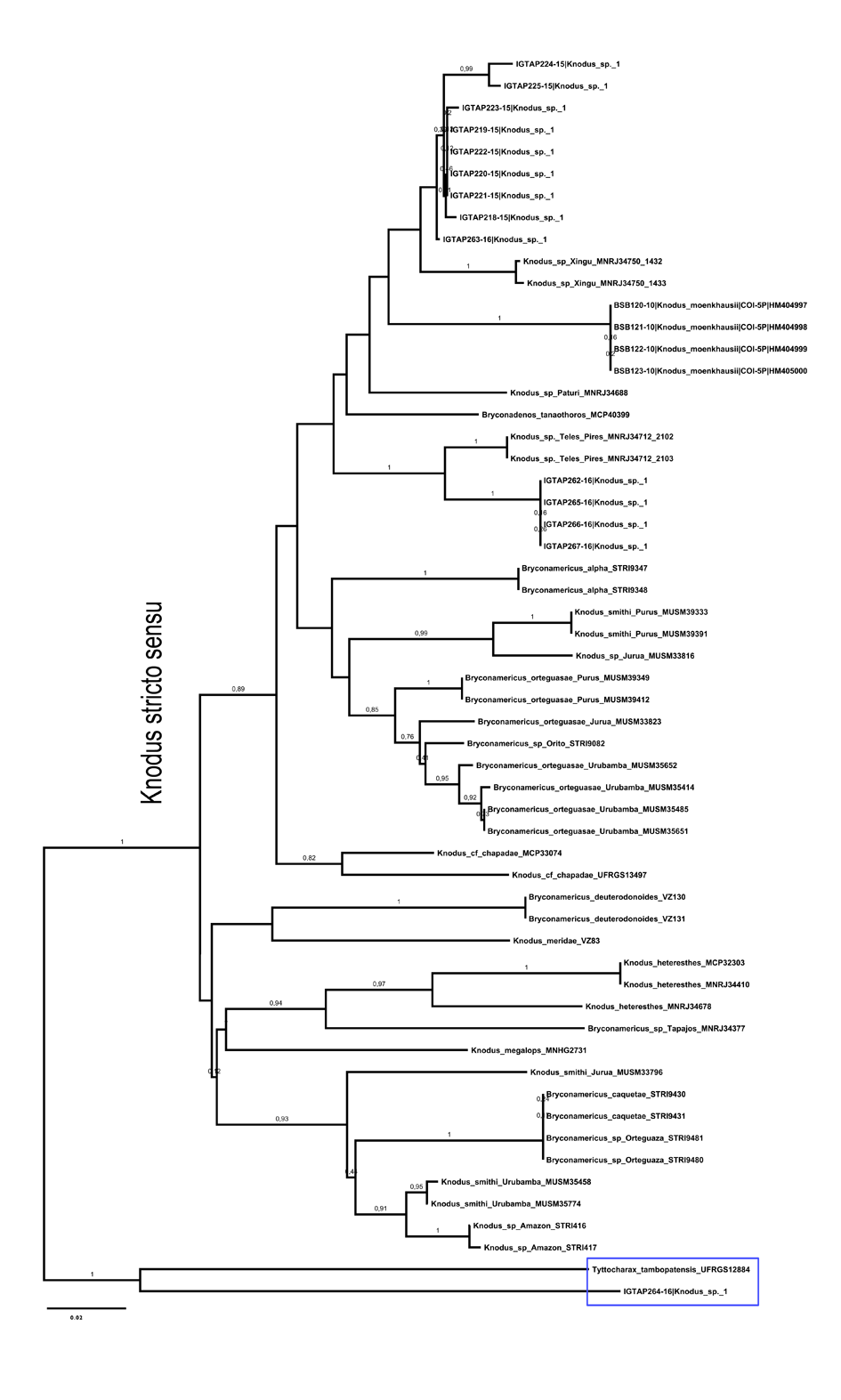

Supplement: S2 Fig — (TIF) [file pone.0209430.s004.tif]
